# Clinical categories of patients and encounter rates in primary health care – a three-year study in defined populations

**DOI:** 10.1186/1471-2458-6-35

**Published:** 2006-02-16

**Authors:** Lennart Carlsson, Lars-Erik Strender, Gerd Fridh, Gunnar H Nilsson

**Affiliations:** 1 Center for Family and Community Medicine, Neurotec Department, Karolinska Institutet, Stockholm, Sweden; 2 Unit for Social Medicine and Health Economics, Stockholm Centre for Public Health, Stockholm County Council, Sweden; 3 Division of Primary Care, Blekinge County Council, Karlskrona, Sweden

## Abstract

**Background:**

The objective was to estimate the proportion of inhabitants with a diagnosis-registered encounter with a general practitioner, and to elucidate annual variations of clinical categories of patients in terms of their individual comorbidity.

**Methods:**

A three-year retrospective study of encounter data from electronic patient records, with an annual-based application of the Johns Hopkins Adjusted Clinical Groups (ACG) system. Data were retrieved from every patient with a diagnosis-registered encounter with a GP during the period 2001–2003 at 13 publicly managed primary health care centres in Blekinge county, southeastern Sweden, with about 150000 inhabitants. Main outcome measures: Proportions of inhabitants with a diagnosis-registered encounter, and ranges of the annual proportions of categories of patients according to ACGs.

**Results:**

The proportion of inhabitants with a diagnosis-registered encounter ranged from about 64.0% to 90.6% for the primary health care centres, and averaged about 76.5% for all inhabitants. In a three-year perspective the average range of categories of patients was about 0.4% on the county level, and about 0.9% on the primary health care centre level. About one third of the patients each year had a constellation of two or more types of morbidity.

**Conclusion:**

About three fourths of all inhabitants had one or more diagnosis-registered encounters with a general practitioner during the three-year period. The annual variation of categories of patients according to ACGs was small on both the county and the primary health care centre level. The ACG system seems useful for demonstrating and predicting various aspects of clinical categories of patients in Swedish primary health care.

## Background

In order to describe and analyse the burden of morbidity in a population, the morbidity and comorbidity status of each patient need to be measured, as well as the mix of groups of patients in a defined area. Case-mix analyses might thus show groups of patients defined by their morbidity status. A case-mix measure classifies *cases *into clinical groups that are similar in terms of certain characteristics. The cases can be patients, contacts, episodes or visits. The characteristics may be diagnosis, procedure, severity, need for resources, and capacity to benefit. Different case-mix systems are constructed to handle different tasks – planning, prevention, describing the content, resource allocation, and cost reimbursement. No single system is applicable to every function [1-3].

Most patient classification systems world-wide have used diagnosis or procedure as the subject for grouping [4-6]. In this study we have focused on patients and their constellation of morbidity. To date, the only system that uses the health status of each patient, and nothing else, as the characteristic for grouping is the Johns Hopkins Adjusted Clinical Groups^® ^(ACG) system [7,8]. It has been designed to predict the need for resources by defined populations and is of particular relevance for studying the health of populations [1]. The objective of the ACG system is to show the burden of morbidity in a population, thereby providing the possibility to allocate resources accordingly [7,8]. The ACG system has been assessed and adapted to the Swedish setting [4,9]. Results from one municipality and one county council have demonstrated the feasibility and the characteristics of the grouping on a one-year basis [10,11]. The ability of the system to elucidate the individual comorbidity of the patients was emphasised. Interest in the ACG system for use in risk management has increased during the past decade, and many academic applications are ongoing world-wide [12-15].

In this study we focus on the first tier of ambulatory care. In Sweden, ambulatory care services outside of hospitals are provided mainly by primary health care (PHC) centres. With very few exceptions no gate-keeping system has been established among the county councils in Sweden, though there often are strong recommendations in many county councils to use PHC as the first level of contact. Diagnoses are classified and coded by the general practitioner (GP), and most PHC centres use electronic patient records (EPRs) [16,17]. The proportion of encounters with a registered diagnosis is about 81% in Swedish PHC [17]. However, when focusing on the morbidity in a population, the proportion of inhabitants with a registered diagnosis is of particular interest. The number of inhabitants with registered diagnoses during one or several years has so far received little attention, and there is to our knowledge no research reported with this focus. The statistics on a national level in Sweden are on resources, procedures and diagnoses, and have not been analysed with respect to the individual constellation of morbidity. However, one study in Sweden with the aim of developing patient-level clinical costing included a limited focus on the constellation of morbidity among patients in PHC [18].

Data retrieval from EPRs on the county level has been done primarily for purposes of disease management and has taken on a traditional epidemiological perspective. To our knowledge, no studies on clinical categories of patients in PHC during a period of several years, in terms of their constellation of morbidity, have been performed in Sweden to date. In the other Scandinavian countries the situation is likewise. With few exceptions, this approach has not even been on the agenda for all of the North American studies aiming to assess and develop the ACG system [19-22].

The aim of this study was to estimate the proportion of inhabitants with at least one encounter where one or more diagnoses are classified and coded by a GP, and to elucidate the annual (year-to-year) variations of clinical categories of patients in PHC by applying the ACG system to encounter data.

## Methods

### Setting and data retrieval

Our study was carried out among all inhabitants of Blekinge county in southeastern Sweden. Data were retrieved from each of the 13 publicly managed PHC centres for the three-year period from 1 January 2001 to 31 December 2003.

When calculating the proportion of inhabitants with a registered diagnosis, we used figures established by the county council for the geographic catchment area of each of the 13 PHC centres. We also assumed that the privately managed PHC units had no geographic catchment area of their own. At the end of 2003 there were 15 such private units.

All of the 13 PHC centres used the Swedestar^® ^EPR system. At three sites two centres combined their EPR databases. Patients at one acute PHC centre were specially registered at that centre. Thus there were 11 sites from which data were retrieved.

Every patient encounter with a GP where there was at least one registered diagnosis was retrieved for each of the three calendar years separately. Four pieces of information were included: an anonymous identification number, date of birth, sex and a diagnostic code.

### Data processing and analysis

The '6.03i' version of the ACG instrument was utilised [23]. The Swedish PHC version of the International Statistical Classification of Diseases and Related Health Problems, Tenth Revision [24], was mapped to the full version of the classification by using a cross-reference scheme based upon equivalent tables from the National Board of Health and Social Services. Detailed information about the ACG system and the grouping algorithms have been published elsewhere [8-11]. The building blocks of the ACGs are the various types of morbidity, where each unique diagnosis (ICD-10) is allotted to one of 32 different ADGs (Aggregated Diagnosis Groups) defining the type of morbidity based on five combined criteria: i) likely persistence of the condition, ii) grade of severity, iii) aetiology, iv) diagnostic certainty and v) need for speciality care. The end result of the grouping is that each patient is allotted to one, and no more than one, of 82 ACGs, depending on his/her registered type or types of morbidity (the ADGs), and/or his or her age and sex in some cases. Thus each ACG is used as an average for a group of patients with the same constellation of ADGs, thereby indicating the need for care of each category of patients.

On the county level the variation within an ACG was calculated with the range of the annual proportion for the three years. On the PHC centre level the variation within an ACG was first calculated in the same way as for the county level for each one of the centres. Then the average range for all PHC centres was calculated. Friedman's test was used to compare the distribution of ACGs at PHC centres between the three years. The statistical software SPSS^® ^version 11.5 was used.

## Results

### Study population

The total population in the county stayed at about the same level during our period of study; 150 017 inhabitants in 2001 and 149 889 in 2003. The total number of patients registered with private GPs amounted to 27 854, which was about 18.6% of all inhabitants at the end of 2003. At the 13 publicly managed PHC centres in Blekinge county there were a total of 119 665 patient encounters with a GP in 2003. On average, 87.1% of the encounters that year had a diagnosis registered, with a range from 78.0% to 96.7% among the PHC centres. The average figure in 2002 was 87.6% and in 2001 it was 89.1%.

The number of patients included in our study, i.e. those patients each year with at least one diagnosis-registered encounter with a GP, decreased from 71 941 in 2001 to 67 212 in 2003. Table 1 gives the number of patients included for each of the participating PHC centres, and also shows the proportion of all inhabitants that were included.

### The proportion of inhabitants with a diagnosis-registered encounter

**Figure 1:**
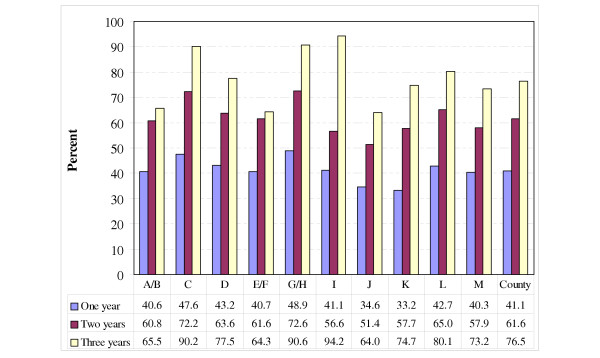
Proportion of inhabitants with a diagnosis-registered encounter with a GP at 13 PHC centres (A-M), and average on the county level for the time periods: calendar year 2003, calendar years 2002–2003 and 2001–2003.

The proportion of inhabitants in the county with a diagnosis-registered encounter with a GP during one calendar year was about 42.7% on average during the three-year period. By identifying each patient on a PHC centre level throughout the three years, it was determined that the proportion of these patients, based on all inhabitants in the county, was about 41.1% during one year (2003), about 61.6% within a two-year perspective (2003 and 2002), and about 76.5% when focusing on all three years (Fig. 1). The proportion of inhabitants, not being registered for a diagnosis by a GP during the three-year period was thus about 23.5%.

### Variation on the county level

**Figure 2:**
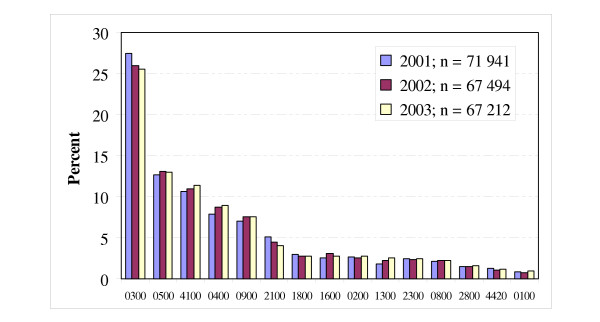
The distribution and three-year range of Adjusted Clinical Groups (ACG) in 2001–2003 (ACG with <1% of the patients were excluded).

Fig. 2 gives an overview for the three years of the distribution of ACGs containing at least 1% of the patients included in our study. The three-year range was 0.4% on average with a maximum of 1.9% in one of the ACGs (Acute: Minor, Age 6+). The 10 most frequent single ACGs comprised about 80% of all patients. Due to incorrect dates of birth, about 30 of the patients included could not be defined in terms of ACGs each year (in total 92 patients, or 0.05%).

**Figure 3:**
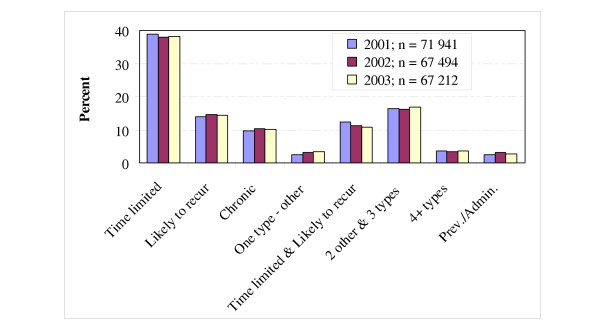
The distribution and three-year average range of Adjusted Clinical Groups (ACG) in 2001–2003, aggregated into eight basic clinical groups.

In Fig. 3 the 82 ACGs have been aggregated into eight clinically based clusters of ACGs. The three-year variation was about 0.5% on average in these clusters, with a maximum of about 1.0%. The lowest range between the three years for an ACG cluster was about 0.1% and the highest value was 1.5%. In 2001 about 38.9% of all patients were categorised as having one and only one *time limited *health condition. The corresponding figures for 2002 and 2003 were 38.0% and 38.2%, respectively. The proportion of patients with a constellation of two or more types of morbidity was 31.5% on average per year.

### Variation on the PHC centre level

On the PHC centre level the average three-year range among all ACGs was about 1.2%, with a maximum of about 3.0%. The average and maximum range for each ACG containing more than 1% of the patients is presented in Table 2. No statistical significant difference in distribution of the ACGs between the three years was found.

## Discussion

The main findings of this study were that three fourths of the inhabitants visited a GP during the three-year period, and a vast majority of the patients had a diagnosis-registered encounter during that period. Also, the annual variation in clinical categories of patients according to ACGs was small in statistical terms. The stability over time provides reasons for using the ACG system both for estimating the proportion of encounters with a GP among the population and for elucidating categories of patients for the purpose of analysing and managing PHC.

One limitation in this study is our assumption that inhabitants living in the geographic catchment area of a particular PHC centre will visit that centre. This has not been analysed, as administrative data were not captured in our study. Further, some patients may also have visited more than one of the 13 PHC centres during a calendar year, and we were not able to trace the patients between centres. Regarding the PHC acute centre, we were able to trace the number of visits of the patients back to their regular PHC centre, but not the number of individuals. Taking these limitations into account, we still consider this county-based approach to be advantageous due to the large population, which probably makes our results fairly representative for PHC in Sweden.

The main limitation of this study is that data were captured only from publicly managed PHC centres. This was due to the lack of EPR systems at the 15 privately managed units. However, less than one fifth of all inhabitants were listed at those units. Further, we believe that a considerable proportion of these patients also visited publicly managed PHC centres. We therefore have reason to believe that this lack of data did not significantly influence the clinical categories of patients in terms of ACGs. Also, there were no differences in access to services between the public and the private PHC units.

The proportion of inhabitants in our study with one or more diagnosis-registered encounters with a GP at the 13 PHC centres during a three-year period (76.5%) seems fairly high, and to our knowledge this has not been studied before. If it had been possible to include encounters at the 15 privately managed units this proportion would doubtless have been larger. Taking into account that not all encounters have a registered diagnosis, the proportion of inhabitants with any encounter with a GP in the three-year period is most likely significantly higher. This indicates that in this respect PHC comprises the first tier of care for the great majority of the population in the county, and our figures are probably representative for PHC in Sweden. Information from EPRs in PHC therefore seems to be useful for further application of the ACG system.

The correctness and completeness of the EPRs and the classification and coding of the diagnoses are important for the ACG system because it is based on the diagnostic codes. The accuracy of the EPRs in general practice in the UK was found to be high for some chronic diseases but poor for acute illness and socio-economic data [17]. However, the retrospective design of our study has given us reliable data in terms of registered diagnoses. The use of everyday clinical data as they were registered in the EPRs has also lead to a fair correctness of the diagnoses. In our study we found a registered diagnosis in about 87% of the encounters, which is in line with another Swedish study [17]. This indicates a fairly high level of completeness, even though about 13% of the total encounters in the council were not diagnosis-registered and thus not included in our study. In the ACG system, however, the crucial point is for the diagnostic code to belong to the right cluster of diagnoses in terms of type of morbidity, based on the five criteria for grouping into the ADGs. This makes the ACG system somewhat less dependent on both the correctness and the completeness of the diagnoses.

Albeit this, the results of the ACG grouping are sensitive to the accuracy with which physicians enter diagnoses into the EPRs. A more complete registration of diagnoses most often leads to a distribution of patient categories with a larger number of complex ACGs.

When measuring the health situation in the populations with the ACG system, the time span of the constellation of morbidity needs to be discussed. The grouping of patients was performed for each year separately, which is so far the only application of the ACG system that has been carried out world-wide. However, in this study we also measured the constellation of morbidity of every individual by means of diagnoses registered during the whole three-year period. On the one hand, this means that more types of morbidity for a patient are taken into account when grouping, resulting in a more comprehensive constellation of morbidity. On the other hand, the morbidity that is present one year might not be relevant the following year. When applying the ACG system to our data from two of the three years in our study, we naturally found that the patients had more complex constellations of morbidity, and this was even more the case when expanding the period to all three years. Further studies on this issue are needed and are ongoing in Blekinge county.

The resulting relatively small three-year variation of ACGs can be considered in two ways. Firstly, it seems that the true distribution of clinical categories of patients measured as ACGs is robust in a three-year perspective, which can be expected due to the large population and the short time span. This might indicate that the ACG system is a valid instrument for measuring the burden of morbidity in a population over time, and might be used for planning purposes by PHC managers. However, further research is needed to analyse the variations in a longer period. Secondly, it seems that the ACG system as an instrument is stable, which has so far received little attention. Our figures on the three-year range (<1%) are low and in line with the results from other studies [25,26]. The ACG system therefore seems to be useful for demonstrating the distribution of, as well as predicting, clinically meaningful categories of patients in Swedish PHC, and it ought to be an interesting area for further research and development.

Additional analyses may also be done on the PHC centre level, such as concerning the constellation of morbidity for various groups of patients with specific diagnoses in a perspective of several years. In the future, the ACG system may provide a basis for more comprehensive analyses such as studies on clinical outcomes.

The resource allocation feature of the ACG system is an important part of the system that has not been specifically addressed in this study; however, the system has been shown to provide a basis for allocating resources [14,20,22,25]. The small variation over time in our study indicates that the ACG system provides a relatively stable basis for allocating resources in PHC. Regarding economic aspects, however, further studies are needed on the performance of the ACG system in Sweden. One trial with this focus was performed with data from an area of Sweden where patient-level clinical costing in PHC has been of interest, and that study is reported on in the following article in this issue of BMC Public Health.

## Conclusion

About three fourths of all inhabitants had one or more diagnosis-registered encounters with a GP during the three-year period. The annual variation of categories of patients according to the ACG system was small on both the county and the PHC centre level. The ACG system therefore seems useful for both demonstrating and predicting the distribution of categories of patients, thereby providing information for the purpose of analysing and managing PHC in Sweden and in other Scandinavian countries.

## Competing interests

The authors declare that they have no competing interests.

## Authors' contributions

LC participated in the design of the study, co-ordinated the work, collected all data, handled the ACG software, performed the analyses, and drafted the manuscript. LES participated in the design of the study and drafted the manuscript. GF checked the data and drafted the manuscript. GHN participated in the design of the study, drafted the manuscript, and helped to co-ordinate the work.

All authors read and approved the final manuscript.

## Pre-publication history

The pre-publication history for this paper can be accessed here:



## Figures and Tables

**Table 1:** Number of patients included in the study at the 13 PHC centres, and the proportion of patients included, based on the total number of inhabitants in the catchment area, in 2001–2003

	2001		2002		2003	
PHC centre	Patients (#)	Prop. of inhabitants (%)	Patients (#)	Prop. of inhabitants (%)	Patients (#)	Prop. of inhabitants (%)

A/B	9 814	43.9	9 012	40.4	9 063	40.4
C	9 007	56.1	8 203	51.2	7 631	47.3
D	2 645	38.8	2 619	38.5	2 939	42.9
E/F	6 950	45.2	6 632	43.2	6 242	40.4
G/H	6 166	52.9	5 798	50.0	5 675	49.2
I	7 557	44.7	7 606	45.2	7 190	42.9
J	5 273	36.3	5 135	35.3	5 980	41.1
K	6 248	38.7	5 853	36.1	5 606	34.6
L	6 480	46.9	5 823	42.8	4 516	33.3
M	7 365	44.9	6 238	38.3	6 568	40.0
N^1^	4 436	--	4 575	--	5 802	--

Total	71 941	45.0^2^	67 494	42.1^2^	67 212	41.0^2^

^1 ^Centre N was a PHC acute centre, and patients came mainly from PHC centres A/B, C and E/F

^2 ^Patients from PHC acute centre N not included

**Table 2:** The three-year average and maximum range of the proportion of Adjusted Clinical Groups (ACGs) at 13 PHC centres in 2001–2003. ACGs with <1% of the patients excluded

ACG	Description	Average range	Maximum range
0300	Acute: Minor, Age 6+	3.0	4.5
0500	Likely to recur, without allergies	1.1	2.3
4100	2–3 Other ADG combinations, Age >34	1.5	2.8
0400	Acute: Major	1.2	2.2
0900	Chronic: Medical, stable	0.8	1.5
2100	Acute: Minor and Likely to recur Age >5, w/out Allergy	1.1	1.9
1800	Acute: Minor, and Acute: Major	0.4	1.1
1600	Preventive/Administrative	1.0	2.1
1300	Psychosocial, without Psychosocial unstable	0.9	1.7

	All ACGs	1.2	3.0

**Table 3:** 

**Range (%)**	**ACG**	**Description**
1.9	0300	Acute: Minor, Age 6+
0.4	0500	Likely to recur, without Allergies
0.7	4100	2–3 Other ADG combinations, Age >34
1.0	0400	Acute: Major
0.6	0900	Chronic: Medical, stable
1.1	2100	Acute: Minor, and Likely to recur, Age >5,w/out Allergy
0.2	1800	Acute: Minor, and Acute: Major
0.6	1600	Preventive/Administrative
0.2	0200	Acute: Minor, Age 2–5
0.7	1300	Psychosocial, without Psychosocial unstable
0.2	2300	Acute: Minor, and Chronic: Medical, stable
0.1	0800	Chronic: Medical, unstable
0.1	2800	Acute: Major, and Likely to recur
0.2	4420	4–5 Other ADG combinations, Age >44, 1 Major ADGs
0.2	0100	Acute: Minor, Age 1

**Table 4:** 

**Basic clinical group**	**Range (%)**	**ACGs included and aggregation principles**
Time limited	0.9	One type of morbidity – time limited
Likely to recur	0.5	One type of morbidity – recurrent
Chronic	0.7	One type of morbidity – chronic
One type – other	0.8	One type of morbidity – others
Time limited & Likely to recur	1.5	Two types of morbidity – time limited and likely to recur
2 other & 3 types	0.6	Two (others than the two above) or three types of morbidity
4+ types	0.3	Four or more types of morbidity
Prev./Admin.	0.6	Preventive/Administrative
